# ECMO management in cardiogenic shock-specialized versus non-cardiogenic shock-specialized centers: a registry-based analysis

**DOI:** 10.1186/s12871-025-03450-y

**Published:** 2025-11-13

**Authors:** Chenglong Li, Xinrui Yu, Liangshan Wang, Xiaomeng Wang, Haixiu Xie, Shuai Zhang, Yiwen Wang, Sheng Zhang, Jianling Liu, Andong Lu, Yan Liu, Yue Huang, Liuer Zuo, Liwen Lyu, Man Huang, Ming Jia, Xing Hao, Feng Yang, Zhongtao Du, Hong Wang, Xiaotong Hou, The Chinese Society of Extracorporeal Life Support (CSECLS) investigators

**Affiliations:** 1 Center for Cardiac Intensive Care, Beijing Anzhen Hospital, Capital Medical University, 2 Anzhen Rd, Chaoyang District, Beijing, 100029 China; 2 Department of Critical Care Medicine, Taizhou Hospital of Zhejiang Province Affiliated to Wenzhou Medical University, Taizhou, Zhejiang China; 3 ICU, The Affiliated Hospital of Guangzhou Medical University, Guangzhou, Guangdong China; 4 Heart Center, The First Hospital of Lanzhou University, Lanzhou, Gansu China; 5 Department of Cardiac Surgery, Wuhan Asia Heart Hospital, Wuhan, Hubei China; 6 ICU, First Affiliated Hospital of Anhui Medical University, Hefei, China; 7 Department of ICU, Shunde Hospital, Southern Medical University, Foshan, Guangdong China; 8 Department of Emergency, The People’s Hospital of Guangxi Zhuang Autonomous Region, Nanning, Guangxi China; 9 Department of General Intensive Care Unit, The Second Affiliated Hospital of Zhejiang University School of Medicine, Hangzhou, Zhejiang China; 10 Beijing Extracorporeal Life Support Quality Control and Improvement Center, Beijing, China

**Keywords:** Cardiogenic shock, Specialized ECMO center, Extracorporeal membrane oxygenation, Mechanical circulatory support, Intra-aortic balloon pump, Critical care outcomes

## Abstract

**Background:**

Data on the impact of center specialization on extracorporeal membrane oxygenation (ECMO) management in cardiogenic shock (CS) remain limited. This study aimed to evaluate differences in outcomes and management of patients with CS receiving ECMO in CS-specialized versus non-CS-specialized centers.

**Methods:**

This registry-based study used data from the Chinese Society of Extracorporeal Life Support (CSECLS) registry. Adult patients diagnosed with CS and treated with ECMO were included. ECMO centers were categorized as CS-specialized or non-specialized based on the responsible department. Propensity score matching (PSM) was conducted to balance patient characteristics and center experience. The primary endpoint was in-hospital mortality.

**Results:**

A total of 1,415 adult patients were included from January 1, 2017, to December 31, 2021 (523 in CS-specialized centers, 892 in non-CS-specialized centers). The mean age was 53.2 ± 16.1 years, and 30.3% of patients were female. In-hospital mortality was lower in CS-specialized centers both before (*P* = 0.001) and after adjustment (*P* = 0.035). Patients in CS-specialized centers more frequently received intra-aortic balloon pumps (38.5% vs. 30.4%; *P* = 0.009), and were less likely to require mechanical ventilation (80.6% vs. 90.7%; *P* < 0.001) or continuous renal replacement therapy (42.7% vs. 49.8%; *P* = 0.030) than those in non-CS-specialized centers.

**Conclusions:**

Treatment in CS-specialized centers was independently associated with lower in-hospital mortality among patients receiving ECMO for circulatory support, even after adjusting for both patient-level characteristics and center-level experience.

**Supplementary Information:**

The online version contains supplementary material available at 10.1186/s12871-025-03450-y.

## Introduction

Cardiogenic shock (CS) is a critical condition characterized by diminished cardiac output and consequent end-organ hypoperfusion, which often leads to multi-organ failure, significant morbidity, and high mortality [1]. In-hospital mortality rates range from 30% to 60%, depending on the underlying etiology [1–4]. Recent studies advocate multidisciplinary team-based strategies for managing CS, particularly involving dedicated CS teams or specialized centers. Within these frameworks, extracorporeal membrane oxygenation (ECMO) has emerged as a pivotal support of patient treatment and recovery when conventional measures fail [5–7].

As a form of temporary support, ECMO provides both circulatory and pulmonary assistance, serving as a bridge to recovery, organ transplantation, permanent mechanical support (such as ventricular assist devices or total artificial heart), and/or decision-making [8]. Originally derived from cardiopulmonary bypass techniques, ECMO has undergone major evolution. Prior to the 2010 s, its primary use was in postcardiotomy cases and acute respiratory failure [9, 10]. Contemporary applications of ECMO extendto a diverse range of clinical scenarios, including acute myocardial infarction complicated by CS, cardiac arrest, fulminant myocarditis, pulmonary embolism, trauma, acute respiratory distress syndrome (ARDS), sepsis, organ transplantation, and other causes of refractory cardiac or respiratory failure [11–14].

With these expanding indications, ECMO is now initiated and managed in diverse settings, not limited to cardiothoracic units, but also including respiratory, medical, surgical, emergency, general intensive care units (ICUs), and prehospital environments as an evolving area of ECMO use [15, 16]. This shift reflects the growing versatility of ECMO application across diverse critical care environments. Although several studies have explored the outcomes of CS patients across different ECMO centers [15], uncertainties remain regarding how care led by CS-specialized units differs from that in non-specialized units, particularly in terms of standardized protocols and ECMO management. Therefore, this study aimed to investigate the differences in management strategies and clinical outcomes of CS patients supported with ECMO in CS-specialized versus non-CS-specialized centers, using data from a national ECMO registry.

## Methods

### Study design and settings

This registry-based study utilized patient data collected from the Chinese Society of Extracorporeal Life Support (CSECLS) registry, with approval from the Central Institutional Review Board (Beijing Anzhen Hospital, Capital Medical University, Beijing, China; approval no. 2019040X). The CSECLS registry records information on ECMO support use, complications, and outcomes at 112 pediatric and adult ECMO centers across China. No personal health information (PHI) was collected, and the requirement for informed consent was waived because patients were not prospectively enrolled specifically for this study. The data acquisition methods have previously been described in detail [17, 18].

**Fig. 1:**
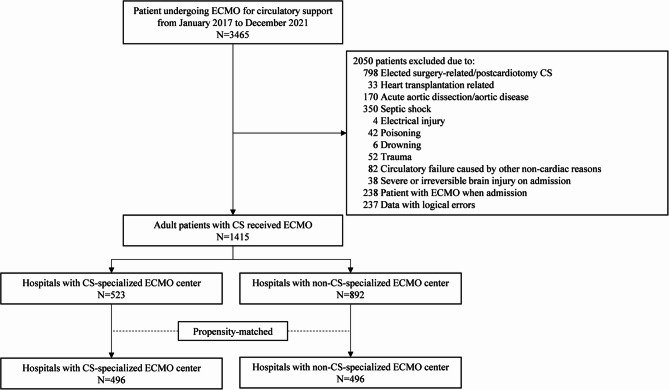
Flow chart of patient selection

Cardiogenic shock in this study was defined according to current guideline criteria [19, 20] as follows: (1) systolic blood pressure (SBP) < 90 mmHg for ≥ 30 min, or the need for vasopressors, inotropes, or mechanical circulatory support to maintain SBP ≥ 90 mmHg; (2) evidence of tissue hypoperfusion, defined as the presence of at least one of the following: elevated arterial lactate (>2 mmol/L), oliguria (< 0.5 mL/kg/h) or acute kidney injury (creatinine ≥ 2×upper limit of normal), acute hepatic injury (ALT >3×upper limit of normal), cool or mottled extremities, or altered mental status not explained by another cause; (3) hemodynamic criteria, including cardiac index ≤ 2.2 L/(min·m^2^) and systemic vascular resistance index >2200 dynes/(cm·sec^−5^). Adult patients (age ≥ 18 years) who were diagnosed with CS during hospitalization and received ECMO support between January 1, 2017, and December 31, 2021, were included. The exclusion criteria were: elective surgery-related CS (e.g., postcardiotomy CS, excluded due to its distinct etiology and standardized postoperative management), heart transplantation, acute aortic dissection/aortic disease, septic shock, electrical injury, poisoning, drowning, trauma, circulatory failure caused by other non-cardiac reasons, severe or irreversible brain injury on admission, ECMO upon admission (transport with ECMO), and datasets with logical errors (Fig. 1). Records with logical errors (e.g., impossible timelines or extreme outliers) were verified with the originating centers and corrected when possible; otherwise, they were excluded from the final analysis. Patients with competing etiologies (e.g., septic shock and trauma) were also excluded.

### ECMO center definition

In the present study, ECMO centers refer to the clinical departments or ICUs primarily responsible for the initiation, management, and decision-making of ECMO support during hospitalization, as recorded in the CSECLS registry. Based on the responsible department, ECMO centers were categorized as either CS-specialized or non-CS-specialized centers. CS-specialized centers included those led by the cardiothoracic ICU (CSICU), coronary care unit (CCU), cardiology department, or cardiothoracic surgery department. These departments specialize in cardiac critical care with dedicated protocols, cardiac surgeons or cardiologists on staff, and 24/7 access to cardiac-specific resources. Non-CS-specialized centers included general ICU, respiratory ICU (RICU), medical ICU (MICU), surgical ICU (SICU), emergency ICUs (EICU), emergency department, anesthesiology, vascular surgery, and extracorporeal circulation departments. Although capable of managing CS patients, these units generally cover a broader spectrum of critical illnesses and often lack dedicated cardiac teams or standardized CS workflows. Centers were classified as “experienced” if they administered > 30 ECMO cases annually and treated > 50 patients during the study period. Department number refers to the number of different clinical departments involved in ECMO management within each center. A higher number indicates that the ECMO team or center is composed of multiple teams and departments. The categorization was uniformly applied across all participating hospitals and is detailed in Table 1. Center-level information regarding hospital-specific characteristics was obtained from official hospital websites.

**Table 1:** ECMO center characteristics

	CS-specialized ECMO center *n* = 29	Non-CS-specialized ECMO center *n* = 47	*P* value
CS cases with ECMO, n	523	892	
Bed capacity, mean (SD)	2995.41(1360.86)	2400.89(1023.95)	0.034
Hospital size, number of beds, n (%)			0.187
< 1000	1(3.4%)	3(6.4%)	
1001–3000	15(51.7%)	31(66.0%)	
3001–5000	21(31.0%)	12(25.5%)	
> 5000	5(13.8%)	1(2.1%)	
Department number^*^, n (%)			< 0.001
1	7(24.1%)	31(66.0%)	
2	5(17.2%)	13(27.7%)	
3	11(37.9%)	3(6.4%)	
4	6(20.7%)	0(0.0%)	
ECMO department, n (%)			
ICU	16(55.2%)	34(72.3%)	0.199
EICU	3(10.3%)	6(12.8%)	1
CCU	5(17.2%)	0(0.0%)	0.014
CSICU	7(24.1%)	0(0.0%)	0.002
RICU	7(24.1%)	4(8.5%)	0.122
SICU	1(3.4%)	1(2.1%)	1
MICU	2(6.9%)	1(2.1%)	0.667
Emergency department	9(31.0%)	7(14.9%)	0.165
Cardiology department	8(27.6%)	0(0.0%)	0.001
Cardiac surgery department	13(44.8%)	0(0.0%)	< 0.001
Anesthesiology department	2(6.9%)	1(2.1%)	0.667
Vascular surgery department	1(3.4%)	0(0.0%)	0.806
Extracorporeal circulation department	0(0.0%)	4(8.5%)	0.278
≥ 2 ECMO center, n(%)	22(75.9%)	16(34.0%)	0.001
Geographic distribution, n(%)			0.011
North	3(10.3%)	4(8.5%)	
Central	5(17.2%)	12(25.5%)	
East	11(37.9%)	13(27.7%)	
South	0(0%)	13(27.7%)	
Southwest	3(10.3%)	3(3.4%)	
Northwest	4(13.8%)	2(4.3%)	
Northeast	3(10.3%)	0(0%)	
Academic hospital, n(%)	27(93.1%)	42(89.4%)	0.702
Experienced centers^*^,n(%)	12(41.4%)	19(40.4%)	0.934

*CS* cardiogenic shock, *ECMO* extracorporeal membrane oxygenation, *ICU* intensive care unit, *EICU* emergency intensive care unit, *CCU* coronary care unit,* MICU* medical intensive care unit, *SICU* surgical intensive care unit, *RICU* respiratory intensive care unit, *CSICU* cardiac surgery intensive care unit

^*^Department number refers to the number of different clinical departments involved in ECMO management within each center

^*^Experienced center refers to ECMO centers administered > 30 ECMO cases per year and treated > 50 patients during the study period

### Clinical parameters and outcomes

Patient characteristics were recorded upon admission. ECMO practice was evaluated as follows: timing of ECMO initiation, i.e., Society for Cardiovascular Angiography and Interventions (SCAI) cardiogenic shock stage or Sequential Organ Failure Assessment (SOFA) score prior to ECMO; ECMO procedure; serum lactate trends; treatments for primary disease (optional items submitted to the database); combined treatments; and ECMO duration. The primary outcome was in-hospital mortality. Secondary outcomes included successful ECMO weaning, complications, duration of mechanical ventilation, length of ICU stay, and length of hospital stay. Surgical site hemorrhage was defined as bleeding from non-cannulation surgical wounds (e.g., thoracic drains or tracheostomy sites).

### Statistical analysis

Continuous variables were presented as mean (standard deviation) or median (interquartile range), and two-group comparisons were performed using Student’s t-test or the Mann-Whitney U test, as appropriate. Categorical variables were compared using Fisher’s exact test. Thirty-day survival after ECMO initiation was estimated via the Kaplan-Meier analysis and compared using the log-rank test. The propensity score was calculated using logistic regression, adjusting for patient characteristics (age, sex, CS etiology, cardiac arrest, comorbidities, prior history, New York Heart Association [NYHA] functional classification, and SCAI stage) and center experience. Based on the propensity scores, patients in CS-specialized centers were 1:1 matched with those in non-CS-specialized centers using the nearest-neighbor method (caliper = 0.2, without replacement). A standardized mean difference (SMD) < 10% indicated an acceptable balance of the measured baseline variables between the two groups. As a sensitivity analysis, a multilevel logistic regression was conducted using generalized linear mixed models, incorporating treatment center as a random effect to account for clustering and unmeasured institutional heterogeneity.

Interaction analyses were performed using logistic regression models in the propensity-matched cohort to assess whether the effect of CS-specialized centers varied across subgroups. The following prespecified subgroups were explored: age ≥ 65; sex; myocarditis; coronary heart disease; acute myocardial infarction; cardiac arrest; NYHA class ≥ III; and SCAI stage. Propensity score matching (PSM) was conducted using the “MatchIt” package in R software 4.2.2 (www.r-project.org). All other analyses were performed using SPSS version 26.0 statistics (IBM Corp., Armonk, NY, USA). A two-sided *P* < 0.05 was considered statistically significant.

## Results

### Patient characteristics

Between January 1, 2017, and December 31, 2021, 76 hospitals contributed data on 3,465 patients who received ECMO for circulatory support to the CSECLS registry. Among these, 1,415 patients with CS met the inclusion criteria (Fig. 1). Of these, 523 (37.0%) were treated in CS-specialized centers, while 892 (63.0%) received care in non-CS-specialized centers. CS-specialized centers were administered by the cardiac ICU (7, 24.1%), coronary care unit (5, 17.2%), cardiac surgery department (13, 44.8%), and cardiology department (8, 27.6%). In contrast, non-CS-specialized centers were more heterogeneous in structure and consisted of the general ICU (34, 72.3%), emergency department or EICUs (13, 27.7%), along with other departments such as medical ICUs, anesthesiology, surgical ICUs, and extracorporeal circulation units. CS-specialized groups more often involved multiple departments (75.9% vs. 34.0%, *P* = 0.001) and were predominantly located in larger hospitals with higher bed capacities (2995 vs. 2401, *P* = 0.034). Detailed characteristics of the ECMO centers are presented in Table 1.

Baseline characteristics are summarized in Table 2. The mean (standard deviation) age was 53.2 ± 16.1 years, and 429 (30.3%) were female. The primary diagnoses of CS patients upon admission were coronary heart disease (55.5%), acute myocardial infarction (48.7%), myocarditis (19.2%), valvular heart disease (6.8%), arrhythmia (6.4%), myopathy (6.3%), and pulmonary embolism (4.1%). A total of 276 (19.5%) patients experienced cardiac arrest before or upon admission.

**Table 2:** Characteristics of patients with cardiogenic shock receiving extracorporeal membrane oxygenation

	Unmatched study cohort			Matched study cohort		
	CS-specialized ECMO center *N* = 523	Non-CS-specialized ECMO center *N* = 892	SMD	CS-specialized ECMO center *N* = 496	Non-CS-specialized ECMO center *N* = 496	SMD
Age, y, mean (SD)	53.2(16.6)	53.3(16.0)	0.009	52.8(16.7)	53.4(16.3)	0.04
Female, n (%)	188(35.9)	241(27.0)	0.193	176(35.5)	176(35.5)	< 0.001
Cardiogenic shock etiology, n (%)						
Myocarditis	100(19.1)	172(19.3)	0.004	99(20.0)	94(19.0)	0.025
Pulmonary embolism	18(3.4)	40(4.5)	0.053	18(3.6)	16(3.2)	0.022
Coronary heart disease	293(56.0)	492(55.2)	0.017	273(55.0)	279(56.2)	0.024
Acute myocardial infarction	260(49.7)	429(48.1)	0.032	243(49.0)	254(51.2)	0.044
Valvular heart disease	37(7.1)	59(6.6)	0.018	35(7.1)	37(7.5)	0.016
Myopathy	48(9.2)	41(4.6)	0.182	42(8.5)	36(7.3)	0.045
Arrhythmia	29(5.5)	62(7.0)	0.058	29(5.8)	32(6.5)	0.025
Others	50(9.6)	95(10.7)	0.036	47(9.5)	45(9.1)	0.014
Cardiac arrest, n (%)	58(11.1)	218(24.4)	0.355	58(11.7)	55(11.1)	0.019
Comorbidities, n (%)						
Hypertension	200(38.2)	320(35.9)	0.049	187(37.7)	192(38.7)	0.021
Hyperlipidemia	58(11.1)	87(9.8)	0.044	53(10.7)	57(11.5)	0.026
Diabetes	110(21.0)	163(18.3)	0.069	103(20.8)	100(20.2)	0.015
Prior history of PCI, n (%)	84(16.1)	138(15.5)	0.016	79(15.9)	76(15.3)	0.017
Prior history of myocardial infarction	78(14.9)	119(13.3)	0.045	72(14.5)	64(12.9)	0.047
NYHA ≥ Class III, n (%)	244(46.7)	361(40.5)	0.125	230(46.4)	240(48.4)	0.040
SCAI stage of CS prior ECMO, n (%)			0.266			0.053
B	65(12.4)	48(5.4)		48(9.7)	43(8.7)	
C	61(11.7)	89(10.0)		59(11.9)	55(11.1)	
D	139(26.6)	248(27.8)		138(27.8)	147(29.6)	
E	258(49.3)	507(56.8)		251(50.6)	251(50.6)	

*PCI* percutaneous coronary intervention, *NYHA* New York Heart Association functional classification, *SCAI* Society for the Cardiovascular Angiography and Interventions shock stage classification, *CS* cardiogenic shock, *ECMO* extracorporeal membrane oxygenation

Before matching, there were more females (35.9% vs. 27.0%; *P* = 0.001), more cases of myopathy (9.2% vs. 4.6%; *P* = 0.001), more NYHA class III/IV patients (46.7% vs. 40.5%; *P* = 0.027), and fewer cardiac arrests (11.1% vs. 24.4%; *P* < 0.001) in the CS-specialized than in the non-CS-specialized group. Patients in the non-CS-specialized group had more severe disease based on the SCAI stage, for example, more stage E (56.8% vs. 49.3%) and fewer stage B (5.4% vs.12.4%) cases (*P* < 0.001). More patients in the CS-specialized ECMO group received ECMO in experienced centers (76.3% vs. 60.3%; *P* < 0.001). After propensity score matching, 496 matched pairs were analyzed. The baseline characteristics considered for the calculation of the propensity score were well balanced between the groups (Table 1**)**. The proportions of patients in experienced centers was 75.0% and 73.4% in the CS-specialized and non-CS-specialized groups, respectively (SMD = 0.037).

### ECMO management

In the unmatched cohort, patients in non-CS-specialized ECMO centers received more extracorporeal cardiopulmonary resuscitation (ECPR; 28.1% vs. 15.5%; *P* < 0.001) and tended to be more severe, as indicated by higher SOFA scores (13 vs. 12; *P* < 0.001) and serum lactate levels before (5.7 vs. 5.6; *P* = 0.001) and 4 h after (8.0 vs. 7.2; *P* = 0.030) ECMO initiation (Table 3). After matching, these differences in the ECPR, SOFA score, and serum lactate levels between the groups were no longer significant. Before and after matching, patients in CS-specialized ECMO centers received more intra-aortic balloon pumps (IABPs; before PSM, 39.2% vs. 29.6%, *P* < 0.001; after PSM, 38.5% vs. 30.4%, *P* = 0.009), including IABPs implanted prior to ECMO (before PSM, 26.4% vs. 18.6%, *P* = 0.001; after PSM, 26.2% vs. 19.4%, *P* = 0.009), had few cases of distal perfusion (before PSM: 48.9% vs. 58.4%, *P* = 0.001; after PSM: 49.8% vs. 58.3%, *P* = 0.009), mechanical ventilation (before PSM: 79.3% vs. 91.0%, *P* < 0.001; after PSM: 80.6% vs. 90.7%, *P* < 0.001), and continuous renal replacement therapy (CRRT; before PSM: 42.3% vs. 48.3%, *P* = 0.031; after PSM: 42.7% vs. 49.8%, *P* = 0.030). Notably, the rate of central cannulation was very low across the entire cohort (before PSM: 0.8% vs. 0.2%, *P* = 0.227; after PSM: 0.8% vs. 0.4%, *P* = 0.682) (Table 3).

**Table 3:** ECMO procedure of patients with cardiogenic shock

	Unmatched study cohort			Matched study cohort		
	CS-specialized ECMO center *N* = 523	Non-CS-specialized ECMO center *N* = 892	P value	CS-specialized ECMO center *N* = 496	Non-CS-specialized ECMO center *N* = 496	P value
ECPR, n (%)	81(15.5)	251(28.1)	< 0.001	80(16.1)	86(17.3)	0.671
Peripheral cannulation^*^, n (%)	519(99.2)	890(99.8)	0.277	492(99.2)	494(99.6)	0.682
Percutaneous methods	372(71.1)	670(75.1)	0.114	352(71.0)	357(72.0)	0.779
Distal perfusion	256(48.9)	520(58.3)	0.001	247(49.8)	289(58.3)	0.009
Central cannulation^*^, n (%)	4(0.8)	2(0.2)	0.277	4(0.8)	2(0.4)	0.682
LV venting, n (%)	28(5.5)	34(4.0)	0.230	28(5.9)	17(3.6)	0.140
Start ECMO during off-hour, n (%)	302(57.7)	561(62.9)	0.063	293(59.1)	323(65.1)	0.058
Time to ECMO initiation, h, (median [IQR])	0.88[0.08,4.41]	0.64[0.07,2.37]	0.005	0.88[0.08,4.15]	0.71[0.11,2.97]	0.955
SOFA score prior to ECMO, mean (SD)	13 [10, 16]	12 [9, 15]	< 0.001	13 [9, 15]	12 [9, 15]	0.055
Serum lactate, mmol/L, mean (SD)						
Prior ECMO	8.11(5.63)	9.13(5.70)	0.001	8.29(5.58)	8.65(5.73)	0.318
4 h after ECMO	7.22(6.41)	8.06(6.55)	0.030	7.48(6.47)	7.69(6.41)	0.630
24 h after ECMO	4.10(4.97)	4.67(5.28)	0.069	4.19(5.05)	4.42(5.15)	0.523
Treatments for primary diseases, n (%)	178/429(41.5)	245/676(36.2)	0.080	162/405(40.0)	130/383(33.9)	0.078
PCI	146(34.0)	218(32.2)	0.539	134(33.1)	113(29.5)	0.279
Cardiac surgery	33(7.7)	32(4.7)	0.042	29(7.2)	20(5.2)	0.260
Combined treatment, n (%)						
Mechanical ventilation	415(79.3)	812(91.0)	< 0.001	400(80.6)	450(90.7)	< 0.001
IABP	205(39.2)	264(29.6)	< 0.001	191(38.5)	151(30.4)	0.009
IABP before ECMO	138(26.4)	166(18.6)	0.001	130(26.2)	96(19.4)	0.012
CRRT	221(42.3)	431(48.3)	0.031	212(42.7)	247(49.8)	0.030
ECMO duration, h, (median [IQR])	95.59 [45.25, 148.69]	94.54 [39.17, 156.76]	0.516	96.00 [40.42, 161.08]	96.00 [47.46, 153.28]	0.78
ECMO duration distribution, n (%)			0.206			0.442
<24 h	87(16.6)	160(17.9)		82(16.5)	87(17.5)	
24–48 h	55(10.5)	111(12.4)		50(10.1)	59(11.9)	
48–120 h	189(36.1)	275(30.8)		178(35.9)	155(31.2)	
>120 h	192(36.7)	346(38.8)		186(37.5)	195(39.3)	

^*^Percutaneous cannulation refers to ECMO insertion via percutaneous puncture without surgical incision or vessel dissection. Central cannulation refers to cannulation of the heart or great vessels, typically performed under open-chest conditions with surgical exposure

*ECPR* extracorporeal cardiopulmonary resuscitation, *ECMO* extracorporeal membrane oxygenation, *SOFA* Sequential Organ Failure Assessment score, *SAVE* Survival After Veno-arterial *ECMO* score, *PCI* percutaneous coronary intervention, *IABP* intra-aortic balloon pump, *CRRT* continuous renal replacement therapy

### Outcomes

In the unmatched cohort, in-hospital mortality was 46.2%, while 1,079 (76.2%) patients were successfully weaned off ECMO. Patients in CS-specialized centers had lower in-hospital mortality rates compared to those in non-CS-specialized centers (40.5% vs. 49.7%, respectively; *P* = 0.001). After matching, patients in CS-specialized centers also had lower in-hospital mortality rates (40.9% vs. 47.6%, respectively; *P* = 0.041; odds ratio [OR] 0.763; 95% confidence interval [CI], 0.594–0.981; *P* = 0.035).

**Fig. 2:**
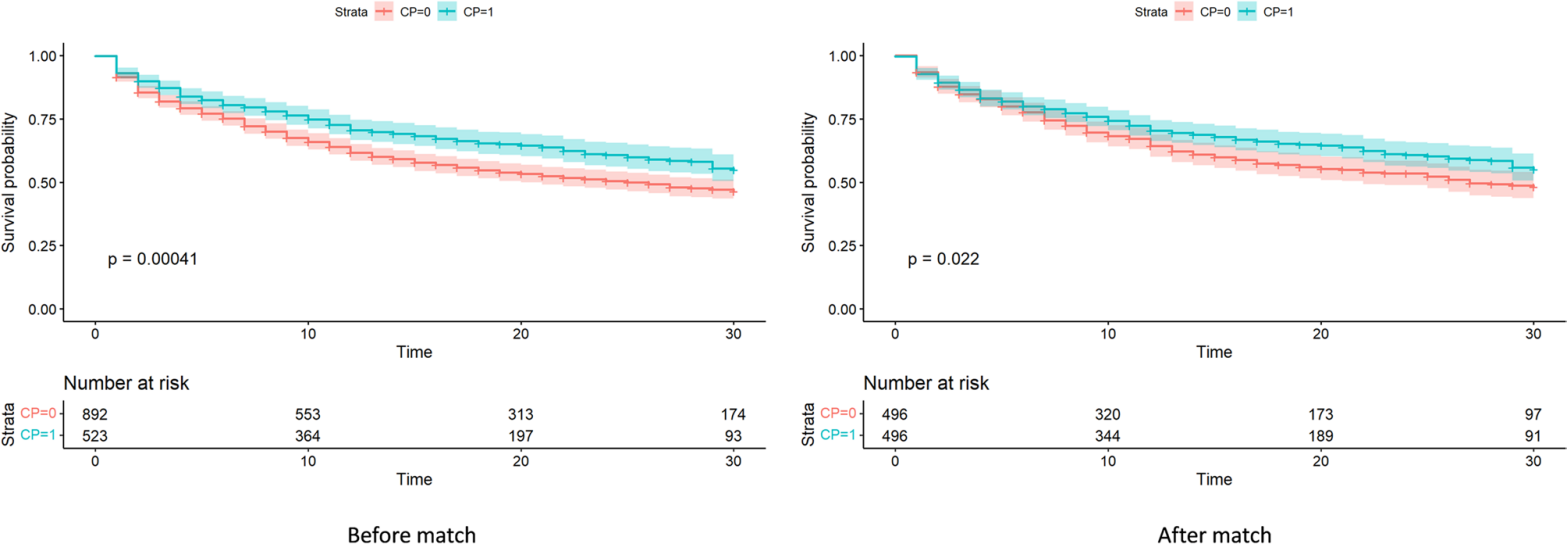
Thirty-day survival curve for cardiogenic shock-specialized versus non-cardiogenic shock-specialized ECMO centers, before and after propensity score matching. CP = 1, cardiogenic shock-specialized center

Regarding secondary outcomes, surgical site bleeding (from non-cannulation wounds, such as thoracic drain or tracheostomy incisions) occurred more frequently in CS-specialized centers compared to non-CS-specialized centers (before PSM: 3.3% vs. 1.2%, *P* = 0.019; after PSM: 3.5% vs. 0.94%, *P* = 0.025). In contrast, a lower incidence of acute kidney injury (AKI; serum creatinine > 3.0 mg/dL) was observed in CS-specialized centers (before PSM: 14.9% vs. 19.5%, *P* = 0.035; after PSM: 14.7% vs. 21.2%, *P* = 0.010). The detailed clinical outcomes are shown in Table 4. Kaplan-Meier analysis demonstrated significantly favorable 30-day survival in the CS-specialized centers in both unmatched (hazard ratio [HR], 0.740; 95% CI, 0.624–0.878; *P* < 0.001) and matched (HR, 0.798; 95% CI, 0.656–0.971; *P* = 0.024) cohorts (Fig. 2).

**Table 4:** Clinical outcomes of patients with cardiogenic shock receiving ECMO

	Unmatched study cohort			Matched study cohort		
	CS-specialized ECMO center *N* = 523	Non-CS-specialized ECMO center *N* = 892	P value	CS-specialized ECMO center *N* = 496	Non-CS-specialized ECMO center *N* = 496	P value
In-hospital mortality, n (%)	212(40.5)	443(49.7)	0.001	203(40.9)	236(47.6)	0.041
Successful weaning from ECMO, n (%)	404(77.2)	675(75.7)	0.544	380(76.6)	382(77.0)	0.940
Vascular injury during cannulation, n (%)	6(1.1)	10(1.1)	1.000	6(1.2)	5(1.0)	1.000
Hemorrhage, n (%)						
Gastrointestinal	21(4.9)	36(4.6)	0.943	19(4.7)	27(6.4)	0.371
Cannulation site	58(13.6)	95(12.2)	0.568	54(13.3)	56(13.2)	1.000
Surgical site	14(3.3)	9(1.2)	0.019	14(3.5)	4(0.9)	0.025
DIC	5(1.2)	14(1.8)	0.546	5(1.2)	6(1.4)	1.000
Neurological complications, n (%)						
Ischemic stroke	7(1.6)	20(2.6)	0.395	7(1.7)	11(2.6)	0.541
Hemorrhagic stroke	7(1.6)	15(1.9)	0.888	6(1.5)	9(2.1)	0.669
Brain death	7(1.6)	17(2.2)	0.659	7(1.7)	4(0.9)	0.492
Nosocomial infection, n (%)	106(25.9)	182(23.6)	0.425	102(26.1)	105(25.1)	0.799
SCr>3.0 mg/dL, n (%)	78(14.9)	174(19.5)	0.035	73(14.7)	105(21.2)	0.010
Hyperbilirubinemia, n (%)	68(13.0)	113(12.7)	0.921	66(13.3)	66(13.3)	1.000
MV duration, hour, (median [IQR])	175.38 [70.30,290.88]	149.06 [48.00,275.00]	0.357	174.69 [68.39,290.06]	149.06 [55.69,289.14]	0.812
ICU length of stay, day, (median [IQR])	10.00 [4.00,17.00]	9.00 [3.00,16.00]	0.109	10.00 [4.00, 17.00]	9.00 [4.00,16.00]	0.275
Hospital length of stay, day, (median [IQR])	14.00 [8.00,26.00]	14.00 [5.00,25.00]	0.076	15.00 [7.00, 26.00]	14.00 [6.00,25.00]	0.467

*ECMO* extracorporeal membrane oxygenation, *DIC* disseminated intravascular coagulation, *SCr* serum creatinine, *MV* mechanical ventilation, *ICU* intensive care unit

**Fig. 3:**
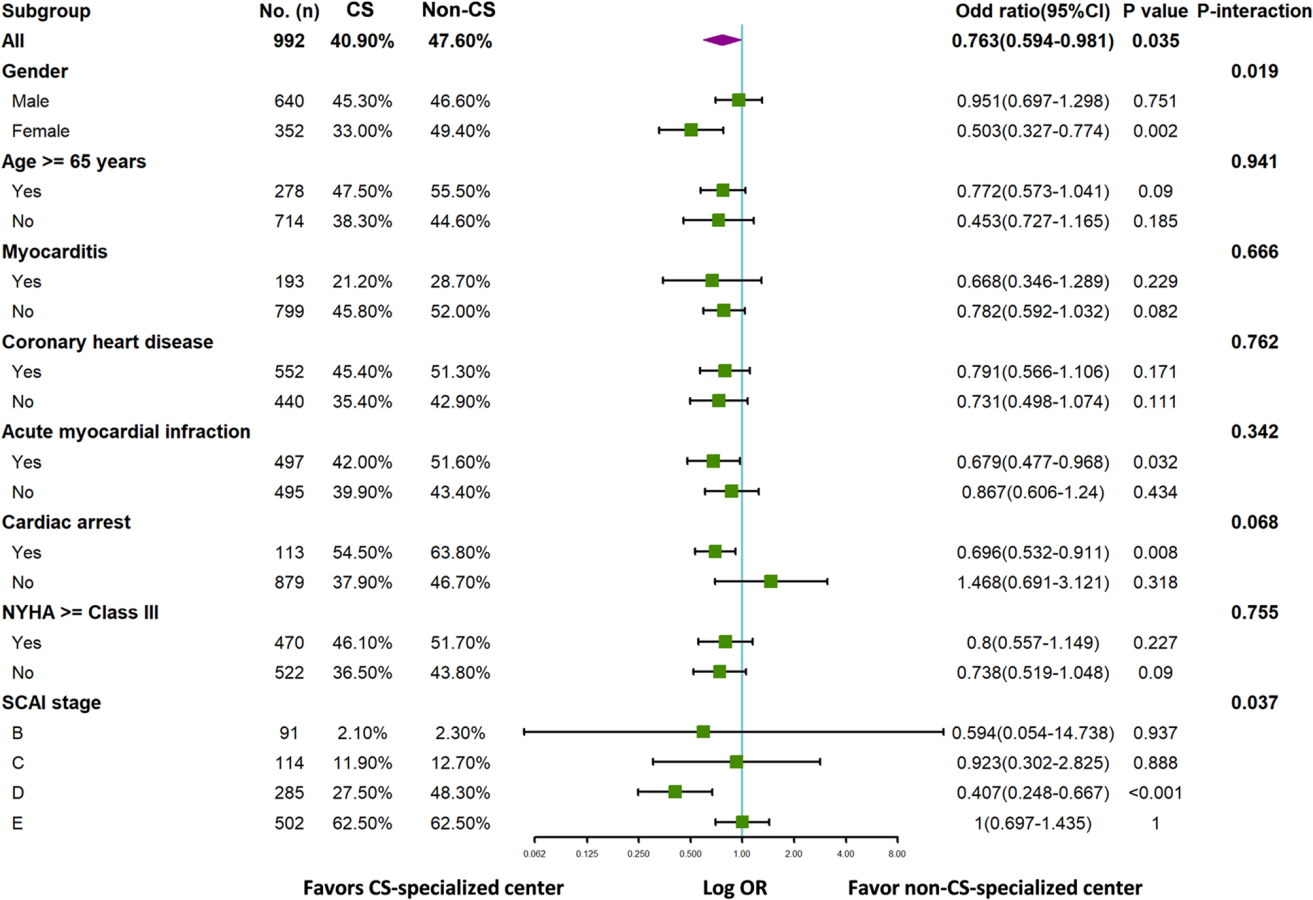
Interaction analysis. NYHA, New York Heart Association functional classification; SCAI, Society for the Cardiovascular Angiography and Interventions shock stage classification

The impact of treatment in CS-specialized centers differed significantly between two subgroups—female and SCAI stage (*P* < 0.05). Subgroup analyses (Fig. 3) revealed significantly stronger associations between CS-specialized care and lower mortality in females (OR, 0.503; 95% CI, 0.327–0.774; *P* = 0.002) and patients with SCAI stage D (OR, 0.407; 95% CI, 0.248–0.667; *P* < 0.001). The interaction P-values for sex and SCAI stage were both < 0.05.

### Analysis in the subset cohort with detailed information on treatments for the primary disease

Considering CS-specialized centers may provide more treatment for primary diseases, an additional analysis was performed in a subset cohort. A total of 1,105 of the 1,415 (78.0%) patients’ data concerning treatments for primary diseases were available in the original cohort. After PSM, the subset cohort contained two well-matched populations of 383 patients each from CS-specialized and non-CS-specialized centers (Tables S1 and S2). The proportion of patients receiving primary disease treatment was slightly higher in the CS-specialized group than in the non-CS-specialized group, but the difference was not statistically significant (41.3% vs. 35.5%, respectively; *P* = 0.106).

In the matched groups, patients in CS-specialized centers also received more IABPs (39.5% vs. 29.6%, respectively; *P* = 0.004), including IABPs implanted before ECMO initiation(26.5% vs. 18.1%; *P* = 0.006), fewer instances of distal perfusion (48.2% vs. 59.2%; *P* = 0.003), mechanical ventilation (77.6% vs. 89.3%; *P* = < 0.001), and CRRT (40.8% vs. 50.0%; *P* = 0.012). The in-hospital mortality rate was slightly lower in the CS-specialized ECMO group but without statistical significance (40.3% vs. 45.7%; *P* = 0.149). No other outcomes differed between the CS-specialized and non-CS-specialized centers (Table S3).

### Multilevel analysis

We conducted a sensitivity analysis using a multilevel logistic regression model with center-specific random intercepts to address potential facility-level confounding. Specifically, treatment in CS-specialized centers remained significantly associated with reduced in-hospital mortality (OR 0.664, 95% CI 0.450–0.982; *P* = 0.040). Other significant predictors included age (OR 1.019 per year; *P* < 0.001), cardiac arrest (OR 0.637; *P* = 0.018), prior myocardial infarction (OR 1.665; *P* = 0.036), and SCAI stage of shock before ECMO initiation. Compared with SCAI stage B, patients in stages D and E had higher odds of in-hospital mortality (OR 22.159 and 69.614; *P* < 0.001). The results (Table S4) were consistent with the primary PSM analysis.

## Discussion

This national registry-based study provides novel insights into the outcomes and management of patients diagnosed with CS who received ECMO in both CS-specialized and non-CS-specialized ECMO settings. The findings revealed that patients in CS-specialized ECMO centers demonstrated lower in-hospital mortality, more frequent use of IABPs, and reduced dependence on mechanical ventilation and CRRT. These mortality differences may reflect earlier recognition of shock, timelier ECMO initiation, greater availability of cardiac-specific interventions, and structured multidisciplinary workflows in CS-specialized centers. Such system-level advantages likely contributed to improved outcomes beyond differences in patient characteristics or ECMO volume alone.

CS is a rapidly progressive, high-mortality condition with diverse etiologies, necessitating prompt diagnosis and multidisciplinary intervention. Recent studies suggest that CS patients benefit from centralized management involving dedicated shock teams or referral to centers equipped with invasive hemodynamic monitors and advanced mechanical circulatory support, including IABPs and ECMO [5, 6]. The present study focused on patients with refractory CS who underwent ECMO and explored how ECMO center type and hospital-level factors influenced clinical strategies and outcomes.

With the evolution of ECMO techniques and indications, the number of hospitals offering ECMO has expanded rapidly, leading to variability in practice and care quality [21]. Notably, only 37% of patients in our cohort were treated in CS-specialized centers, indicating that a substantial portion of ECMO support for CS is still delivered in non-CS-specific settings, where specialized expertise and protocolized care may be lacking. This distribution likely reflects the real-world growth of ECMO programs in general hospitals, where CS patients are often managed by non-specialized teams due to logistical or regional constraints. In the unmatched cohort, we observed a 9% absolute reduction in in-hospital mortality among patients treated in CS-specialized centers.

In high-resource healthcare systems, it is well established that CS patients benefit from care in specialized centers with dedicated heart failure and mechanical circulatory support [5]. However, in many developing regions or newly established ECMO teams, ECMO is often initiated in general ICUs without support from specialized cardiac teams. In these non-specialized settings, ECMO care is typically provided without interdisciplinary collaboration or standardized protocols, potentially contributing to delays in decision-making, inconsistent patient selection, and fragmented post-ECMO care—all of which may adversely impact outcomes.

Our study underscores this concern and confirms that ECMO centers anchored within cardiac specialties, such as cardiac surgery, cardiology, or cardiac intensive care, are associated with better survival outcomes. This supports the value of establishing standardized CS workflows, enhancing interdepartmental collaboration, and investing in personnel training to improve patient outcomes.

A noteworthy observation was the relatively high proportion of patients classified as SCAI Stage B or C, particularly in CS-specialized centers. Although ECMO is traditionally reserved for patients in refractory shock (SCAI Stage D or E) [22], early ECMO initiation (i.e., SCAI Stage B or C) is increasingly advocated to prevent irreversible organ injury and facilitate recovery in cases of rapidly deteriorating myocardial infarction, myocarditis, or pharmacologic treatment failure [23–25]. The higher representation of early-stage CS patients in CS-specialized centers may reflect evolving clinical strategies. In this context, ECMO is a component of a salvage strategy to halt disease progression, which may partly explain the improved outcomes observed in these centers.

Differences in baseline characteristics were evident between the groups, including fewer cardiac arrests, fewer SCAI stage E presentations, a higher proportion of female, and more patients with myopathy in the CS-specialized group. Several factors may explain these findings. First, CS-specialized centers likely recognize CS earlier and escalate to MCS timelier than non-CS centers, facilitated by dedicated teams and standard shock protocols, resulting in less severe conditions. Second, referral patterns may account for the higher prevalence of myopathy, as patients with cardiomyopathies (e.g., dilated, hypertrophic, or genetic etiologies) are more often admitted directly to cardiac ICUs, rather than general ICUs or emergency departments. These units are more familiar with managing such complex conditions, leading to their over-representation in the CS-specialized group. Third, the higher proportion of female patients may reflect unmeasured differences in referral or possibly more proactive treatment strategies for females with acute cardiac conditions in CS-specialized centers. Prior studies have shown sex disparities in shock management, and specialized centers may narrow this gap through structured care [26]. Additionally, considering that the hospital-level volume of ECMO cases correlates with mortality, center experience was also a focus of the present study [21]. After adjusting for baseline characteristics and center experience, a 1:1 matched population analysis demonstrated lower in-hospital mortality in CS-specialized centers (40.9% vs. 47.6%).

The difference in hospital survival may be attributed to variations in ECMO management strategies. The timing of ECMO initiation was adjusted through PSM, and in the matched cohort, the patients’ conditions, such as pre-ECMO serum lactate level and SOFA score, were comparable between groups. Time to ECMO initiation, defined as the interval from hospital admission or recognition of cardiogenic shock to ECMO cannulation, has been used as a general indicator of support timeliness [25]. Both groups had a median time-to-ECMO-initiation of 0.8 h, with no significant difference. Notably, IABP use was more frequent in CS-specialized centers (38.5% vs. 30.4%). Although the benefit of IABP use in CS remains controversial [27, 28], it is still the primary strategy for left ventricular unloading during ECMO. Recent evidence from the CSECLS registry has demonstrated that survival benefit is associated with IABP use post-ECMO initiation among CS patients [29]. Similarly, data from the Extracorporeal Life Support Organization (ELSO) indicated improved survival with left ventricular unloading, albeit with higher risks of bleeding and hemolysis [30]. This may partially account for the higher hemorrhagic event rate in the CS-specialized group. The proportion of central cannulation in our cohort was relatively low. This is consistent with current practice patterns across most centers, where peripheral cannulation is preferred due to its rapid deployment, lower invasiveness, and feasibility. Central cannulation is generally reserved for specific scenarios such as intraoperative failure to wean from CPB. Since elective PCS cases were excluded from our study, the overall frequency of central cannulation was further reduced.

Additionally, patients in CS-specialized centers required less mechanical ventilation and CRRT. Recent studies have linked awake ECMO management to a lower incidence of pneumonia and improved short- and long-term outcomes [31, 32]. Furthermore, the relationship between AKI, CRRT use, and increased mortality is well-established [33, 34], consistent with our findings. After balancing patient characteristics, the type of ECMO center appears to play a key role in combined treatment strategies.

In the unmatched cohort, a higher proportion of treatments for primary disease was observed in the CS-specialized centers both before (41.5% vs. 36.2%) and after (40.0% vs. 33.9%) PSM, likely due to the properties of these centers. In the subset of patients with detailed treatment data, the CS-specialized group continued to show higher intervention rates (41.3% vs. 35.5%) and lower in-hospital mortality (40.3% vs. 45.7%), though the difference was not significant. Existing evidence supports the benefit of disease-specific interventions such as percutaneous coronary intervention (PCI) in CS [35, 36]. The present study is the first to describe PCI and cardiac surgery for primary diseases in a mixed ECMO-CS population [3, 37, 38]. The treatment of primary diseases may be a crucial factor in improving outcomes in ECMO-supported CS, warranting further investigation.

We also explored the impact of CS-specialized centers on clinical outcomes in specific subgroups. In female patients and those classified as SCAI stage D, CS-specialized care was significantly associated with improved in-hospital survival. Prior literature indicates that the female sex is independently linked to higher in-hospital mortality in CS, potentially due to poorer physical condition, fewer cardiac procedures, and more complications [26, 39]. Therefore, the therapeutic effects may be more pronounced in females. Previous studies have shown that mortality risk increases with the initial SCAI stage [37, 40]. Early SCAI shock stages (A, B, and C) are generally easier to recognize and manage; as a result, the added value of CS-specialized centers may be less pronounced in these patients. In contrast, patients who progress to SCAI stage D—characterized by hemodynamic compromise—are more likely to benefit from transfer to a multidisciplinary CS-specialized center, where comprehensive heart failure management is available [41]. Moreover, prior studies have reported that the in-hospital mortality rate for SCAI stage E exceeds 67% [42, 43], highlighting that patients in such advanced stages may derive limited benefit from even aggressive interventions due to irreversible end-organ damage.

Another notable finding was the higher frequency of surgical site hemorrhage in the CS-specialized group, observed in both the unmatched and matched cohorts. The present study illustrated that the major risk factor for bleeding in patients with CS was receiving mechanical circulatory support (including IABP and ECMO), which induces high shear stress on blood elements and necessitates intensive anticoagulation therapy [44]. Patients in the CS-specialized centers tended to undergo more aggressive interventions, which may potentially elevate the risk of bleeding—a pattern consistent with recent meta-analytic evidence [45]. Additionally, a higher proportion of patients in the non-CS-specialized group had serum creatinine levels ≥ 3.0 mg/dL, a marker strongly associated with increased mortality. Early recognition and management of CS before the development of severe AKI may contribute to reduced mortality and the subsequent need for CRRT [5].

To further validate our findings and account for inter-hospital heterogeneity, we conducted multilevel logistic regression analyses incorporating center-level random effects. This statistical approach adjusts for both patient-level covariates and institutional clustering, thereby offering a more accurate estimation of program-level impact in a multicenter registry. Notably, the CS-specialized ECMO team remained independently associated with reduced in-hospital mortality even after adjusting for between-center variation. This supports the robustness of our conclusions and underscores the importance of center specialization and structured design in improving outcomes, beyond individual characteristics.

### Limitations

There are several limitations in this study. First, as a registry-based study involving multiple centers, residual confounding and unmeasured variables could not be fully excluded despite using PSM. Second, patients with cardiac surgery- and heart transplantation-related CS were excluded from the analysis, which may limit the generalizability of our conclusions, as such patients are typically not admitted to non-CS departments. Third, the absence of post-discharge follow-up data precludes the assessment of long-term outcomes differences between the two center types.

Although the current analysis cannot establish a causal relationship due to its registry-based design, it contributes to the growing body of evidence supporting the potential benefits of CS-specialized centers in managing CS. Given the global surge in the utilization of ECMO and the establishment of ECMO centers across various departments, our findings underscore that CS-specialized ECMO centers may be associated with improved in-hospital outcomes. Nevertheless, the challenge remains to ensure early identification, standardized treatment pathways, coordinated multidisciplinary care, and minimized variability in practice across ECMO centers in CS management.

## Conclusions

Treatment in cardiogenic shock (CS)-specialized centers was independently associated with lower in-hospital mortality among patients receiving ECMO for circulatory support, even after adjusting for both patient-level characteristics and center-level experience.

## Supplementary Information

Supplementary Material 1.

## Data Availability

The datasets used and analyzed during the current study are available from the corresponding author on reasonable request.

## Abbreviations

CSECLS: The Chinese society of extracorporeal life support
ECMO: Extracorporeal membrane oxygenation
CS: Cardiogenic shock
ARDS: Acute respiratory distress syndrome
ICUs: Intensive care units
PHI: Personal health information
SCAI: Society for the Cardiovascular Angiography and Interventions Shock Stage Classification
SOFA: Sequential Organ Failure Assessment score
NYHA: New York Heart Association functional classification
SMD: Standardized mean difference
PSM: Propensity score matching
ECPR: Extracorporeal cardiopulmonary resuscitation
CPB: Cardiopulmonary bypass
IABP: Intra-aortic balloon pump
CRRT: Continuous renal replacement therapy
OR: Odds ratio
CI: Confidence interval
AKI: Acute kidney injury
HR: Hazard ratio
PCI: Percutaneous coronary intervention
SAVE: Survival After Veno-arterial ECMO score
DIC: Disseminated intravascular coagulation
SCr: Serum creatinine
MV: Mechanical ventilation
