# Supplementary material for: ECMO management in cardiogenic shock-specialized versus non-cardiogenic shock-specialized centers: a registry-based analysis
Source: BMC Anesthesiol. 2025 Nov 13;25:557. doi: 10.1186/s12871-025-03450-y (PMC12613356; doi:10.1186/s12871-025-03450-y)
Supplement: Supplementary file 1 — Supplementary Material 1. [file 12871_2025_3450_MOESM1_ESM.docx]

**Table S1** Characteristics of patients with cardiogenic shock receiving extracorporeal membrane oxygenation

|  | Unmatched study cohort | | | Matched study cohort | | |
| --- | --- | --- | --- | --- | --- | --- |
|  | CS-specialized  N=429 | Non-CS-specialized  N=676 | SMD | CS-specialized  N=383 | Non-CS-specialized  N=383 | SMD |
| Age, y, mean (SD) | 52.3(16.9) | 52.5(16.4) | 0.011 | 52.3(16.9) | 52.3(16.8) | 0.003 |
| Female, n (%) | 163(38.0) | 197(29.1) | 0.188 | 147(37.5) | 160(40.8) | 0.068 |
| Cardiogenic shock etiology, n (%) |  |  |  |  |  |  |
| Myocarditis | 98(22.8) | 172(25.4) | 0.061 | 94(24.0) | 90(23.0) | 0.024 |
| Pulmonary embolism | 10(2.3) | 22(3.3) | 0.056 | 10(2.6) | 8(2.0) | 0.034 |
| Coronary heart disease | 220(51.3) | 342(50.6) | 0.014 | 197(50.3) | 204(52.0) | 0.036 |
| Acute myocardial infarction | 197(45.9) | 307(45.4) | 0.01 | 179(45.7) | 188(48.0) | 0.046 |
| Valvular heart disease | 35(8.2) | 57(8.4) | 0.01 | 34(8.7) | 33(8.4) | 0.009 |
| Myopathy | 45(10.5) | 41(6.1) | 0.161 | 38(9.7) | 37(9.4) | 0.009 |
| Arrhythmia | 26(6.1) | 44(6.5) | 0.018 | 26(6.6) | 28(7.1) | 0.02 |
| Others | 38(8.9) | 48(7.1) | 0.065 | 30(7.7) | 28(7.1) | 0.019 |
| Cardiac arrest, n (%) | 37(8.6) | 132(19.5) | 0.317 | 37(9.4) | 42(10.7) | 0.042 |
| Comorbidities, n (%) |  |  |  |  |  |  |
| Hypertension | 155(36.1) | 222(32.8) | 0.069 | 141(36.0) | 133(33.9) | 0.043 |
| Hyperlipidemia | 37(8.6) | 63(9.3) | 0.024 | 33(8.4) | 34(8.7) | 0.009 |
| Diabetes | 91(21.2) | 112(16.6) | 0.119 | 75(19.1) | 85(21.7) | 0.063 |
| Prior history of PCI, n (%) | 65(15.2) | 104(15.4) | 0.006 | 61(15.6) | 59(15.1) | 0.014 |
| Prior history of myocardial infarction | 54(12.6) | 84(12.4) | 0.005 | 44(11.2) | 49(12.5) | 0.039 |
| NYHA ≥ Class III, n (%) | 212(49.4) | 247(36.5) | 0.262 | 185(47.2) | 181(46.2) | 0.02 |
| SCAI stage of CS prior ECMO, n (%) |  |  | 0.214 |  |  | 0.045 |
| B | 52(12.1) | 41(6.1) |  | 42(10.7) | 37(9.4) |  |
| C | 50(11.7) | 78(11.5) |  | 46(11.7) | 46(11.7) |  |
| D | 119(27.7) | 206(30.5) |  | 112(28.6) | 116(29.6) |  |
| E | 208(48.5) | 351(51.9) |  | 192(49.0) | 193(49.2) |  |

PCI percutaneous coronary intervention, NYHA New York Heart Association functional classification, SCAI Society for the Cardiovascular Angiography and Interventions shock stage classification, CS cardiogenic shock, ECMO extracorporeal membrane oxygenation

**Table S2** ECMO procedure of patients with cardiogenic shock

|  | Unmatched study cohort | | | Matched study cohort | | |
| --- | --- | --- | --- | --- | --- | --- |
|  | CS-specialized  N=429 | Non-CS-specialized  N=676 | P value | CS-specialized  N=383 | Non-CS-specialized  N=383 | P value |
| ECPR, n (%) | 51(11.9) | 147(21.7) | <0.001 | 50(12.8) | 62(15.8) | 0.262 |
| Peripheral cannulation, n (%) | 388(90.4) | 589(87.1) | 0.114 | 353(90.1) | 340(86.7) | 0.181 |
| Percutaneous methods | 298(69.5) | 499(73.8) | 0.133 | 274(69.9) | 292(74.5) | 0.175 |
| Distal perfusion | 204(47.6) | 393(58.1) | 0.001 | 189(48.2) | 232(59.2) | 0.003 |
| Central cannulation, n (%) | 4(0.9) | 1(0.1) | 0.152 | 3(0.8) | 1(0.3) | 0.616 |
| LV venting, n (%) | 24(5.8) | 22(3.4) | 0.096 | 23(6.1) | 12(3.2) | 0.097 |
| Start ECMO during off-hour, n (%) | 250(58.3) | 423(62.6) | 0.173 | 232(59.2) | 252(64.3) | 0.163 |
| Time to ECMO initiation, h, (median [IQR]) | 0.90 [0.10, 4.31] | 0.62 [0.07, 2.68] | 0.010 | 0.84 [0.08, 4.01] | 0.70 [0.10, 3.00] | 0.573 |
| SOFA score prior to ECMO, mean (SD) | 12 [9, 15] | 13 [9, 16] | <0.001 | 12 [9, 15] | 12 [9, 15] | 0.185 |
| Serum lactate, mmol/L, mean (SD) |  |  |  |  |  |  |
| Prior ECMO | 7.88(5.29) | 8.80(5.67) | 0.007 | 8.02(5.30) | 8.58(5.81) | 0.155 |
| 4h after ECMO | 7.00(6.20) | 7.77(6.54) | 0.073 | 7.10(6.27) | 7.82(6.68) | 0.150 |
| 24h after ECMO | 3.98(4.68) | 4.48(5.26) | 0.145 | 4.10(4.79) | 4.56(5.30) | 0.241 |
| Treatments for primary diseases, n (%) | 178(41.5) | 245(36.2) | 0.092 | 162(41.3) | 139(35.5) | 0.106 |
| PCI | 146(34.0) | 218(32.2) | 0.583 | 133(33.9) | 123(31.4) | 0.493 |
| Cardiac surgery | 33(7.7) | 32(4.7) | 0.057 | 30(7.7) | 20(5.1) | 0.188 |
| Combined treatment, n (%) |  |  |  |  |  |  |
| Mechanical ventilation | 328(76.5) | 608(89.9) | <0.001 | 304(77.6) | 350(89.3) | <0.001 |
| IABP | 171(39.9) | 196(29.0) | <0.001 | 155(39.5) | 116(29.6) | 0.004 |
| IABP before ECMO | 116(27.0) | 126(18.6) | 0.001 | 104(26.5) | 71(18.1) | 0.006 |
| CRRT | 175(40.8) | 323(47.8) | 0.027 | 160(40.8) | 196(50.0) | 0.012 |
| ECMO duration, h, (median[IQR]) | 98.37 [49.00, 149.00] | 99.00 [43.74, 161.46] | 0.984 | 102.00 [49.00, 150.25] | 105.34 [47.49, 165.82] | 0.703 |
| ECMO duration distribution, n (%) |  |  | 0.182 |  |  | 0.674 |
| <24 h | 63(14.7) | 103(15.2) |  | 57(14.5) | 61(15.6) |  |
| 24-48 h | 45(10.5) | 84(12.4) |  | 40(10.2) | 40(10.2) |  |
| 48-120 h | 158(36.8) | 207(30.6) |  | 144(36.7) | 128(32.7) |  |
| >120 h | 163(38.0) | 282(41.7) |  | 151(38.5) | 163(41.6) |  |

ECPR extracorporeal cardiopulmonary resuscitation, ECMO extracorporeal membrane oxygenation, SOFA Sequential Organ Failure Assessment score, SAVE Survival After Veno-arterial ECMO score, PCI percutaneous coronary intervention, IABP intra-aortic balloon pump, CRRT continuous renal replacement therapy

**Table S3** Clinical outcomes of patients with cardiogenic shock receiving ECMO

|  | Unmatched study cohort | | | Matched study cohort | | |
| --- | --- | --- | --- | --- | --- | --- |
|  | CS-specialized  N=429 | Non-CS-specialized  N=676 | P value | CS-specialized  N=383 | Non-CS-specialized  N=383 | P value |
| In-hospital mortality, n (%) | 169(39.4) | 319(47.2) | 0.013 | 158(40.3) | 179(45.7) | 0.149 |
| Successful weaning from ECMO, n (%) | 337(78.6) | 510(75.4) | 0.263 | 306(78.1) | 292(74.5) | 0.275 |
| Vascular injury during cannulation, n (%) | 2(0.5) | 10(1.5) | 0.199 | 1(0.3) | 5(1.3) | 0.219 |
| Hemorrhage, n (%) |  |  |  |  |  |  |
| Gastrointestinal | 13(3.8) | 27(4.7) | 0.65 | 13(4.3) | 21(6.6) | 0.271 |
| Cannulation site | 51(15.1) | 76(13.3) | 0.51 | 44(14.5) | 47(14.8) | 1.000 |
| Surgical site | 12(3.6) | 9(1.6) | 0.091 | 12(3.9) | 7(2.2) | 0.302 |
| DIC | 3(0.9) | 7(1.2) | 0.888 | 3(1.0) | 2(0.6) | 0.960 |
| Neurological complications, n (%) |  |  |  |  |  |  |
| Ischemic stroke | 6(1.8) | 18(3.1) | 0.301 | 6(2.0) | 7(2.2) | 1.000 |
| Hemorrhagic stroke | 5(1.5) | 14(2.4) | 0.455 | 5(1.6) | 9(2.8) | 0.468 |
| Brain death | 6(1.8) | 11(1.9) | 1 | 6(2.0) | 4(1.3) | 0.696 |
| Nosocomial infection, n (%) | 89(27.5) | 134(23.7) | 0.239 | 79(26.9) | 84(26.8) | 1.000 |
| SCr＞3.0 mg/dL, n (%) | 69(16.1) | 139(20.6) | 0.076 | 65(16.6) | 86(21.9) | 0.070 |
| Hyperbilirubinemia, n (%) | 63(14.7) | 85(12.6) | 0.361 | 58(14.8) | 56(14.3) | 0.919 |
| MV duration, hour, (median [IQR]) | 176.56[76.16, 291.28] | 176.56[76.16, 291.28] | 0.633 | 177.75[76.33, 295.55] | 161.86 [88.25, 292.00] | 0.994 |
| ICU length of stay, day, (median [IQR]) | 10.00[5.00, 17.00] | 10.00[4.00, 17.00] | 0.436 | 10.00[5.00, 17.25] | 9.50 [4.00, 17.00] | 0.602 |
| Hospital length of stay, day, (median [IQR]) | 14.00[8.00, 26.00] | 14.00 [6.00, 26.00] | 0.744 | 15.00[8.00, 26.00] | 14.00[7.00, 27.25] | 0.841 |

ECMO extracorporeal membrane oxygenation, DIC disseminated intravascular coagulation, SCr serum creatinine, MV mechanical ventilation, ICU intensive care unit

**Table S4** Multilevel logistic regression analysis of factors associated with in-hospital mortality

|  | Odd Ratio | 95% Confidence Interval | P value |
| --- | --- | --- | --- |
| CS-specialized center, n (%) | 0.664 | 0.450-0.982 | 0.040 |
| Experienced centers, n (%) | 1.220 | 0.839-1.773 | 0.299 |
| Age, y, mean (SD) | 1.019 | 1.009-1.029 | <0.001 |
| Female, n (%) | 0.897 | 0.677-1.188 | 0.448 |
| Cardiogenic shock etiology, n (%) |  |  |  |
| Myocarditis | 0.229 | 0.107-0.486 | <0.001 |
| Pulmonary embolism | 0.580 | 0.240-1.398 | 0.225 |
| Coronary heart disease | 0.583 | 0.276-1.230 | 0.157 |
| Acute myocardial infarction | 0.844 | 0.481-1.479 | 0.552 |
| Valvular heart disease | 0.676 | 0.330-1.385 | 0.284 |
| Myopathy | 0.680 | 0.310-1.489 | 0.334 |
| Arrhythmia | 0.880 | 0.508-1.523 | 0.647 |
| Others | 0.920 | 0.431-1.961 | 0.829 |
| Cardiac arrest, n (%) | 0.637 | 0.438-0.925 | 0.018 |
| Comorbidities, n (%) |  |  |  |
| Hypertension | 0.984 | 0.728-1.330 | 0.917 |
| Hyperlipidemia | 1.181 | 0.757-1.842 | 0.464 |
| Diabetes | 0.855 | 0.610-1.198 | 0.362 |
| Prior history of PCI, n (%) | 0.759 | 0.498-1.156 | 0.199 |
| Prior history of myocardial infarction | 1.665 | 1.034-2.681 | 0.036 |
| NYHA ≥ Class III, n (%) | 1.084 | 0.817-1.437 | 0.577 |
| SCAI stage of CS prior ECMO, n (%) |  |  |  |
| B | (reference) |  |  |
| C | 4.363 | 1.391-13.685 | 0.012 |
| D | 22.159 | 7.797-62.975 | <0.001 |
| E | 69.614 | 24.510-197.720 | <0.001 |
| (Intercept) | 0.030 | 0.007-0.123 | <0.001 |

ECMO extracorporeal membrane oxygenation, CS cardiogenic shock, SCAI Society for Cardiovascular Angiography and Interventions, NYHA New York Heart Association, PCI percutaneous coronary intervention
